# Study Design of Draw‐10: a weekly blood draw study to understand the biological fluctuations of plasma pTau‐217

**DOI:** 10.1002/alz70856_100492

**Published:** 2025-12-25

**Authors:** Ricquee Gonzalez‐Green, Trisha L Walsh, Claire M Erickson, Nellie M. High, Carolyn Langlois, Kari Dieckhoff, James Liu, Marisa N. Denkinger, Kaj Blennow, Wagner Scheeren Brum, Eric M. Reiman, Nicholas J. Ashton, Jessica B. Langbaum

**Affiliations:** ^1^ Banner Alzheimer's Institute, Phoenix, AZ, USA; ^2^ Banner Sun Health Research Institute, Sun City, AZ, USA; ^3^ Department of Psychiatry and Neurochemistry, Institute of Neuroscience and Physiology, The Sahlgrenska Academy, University of Gothenburg, Mölndal, Sweden

## Abstract

**Background:**

Blood biomarkers are becoming pivotal tools in the diagnosis and management of neurodegenerative diseases, such as Alzheimer's disease (AD). The leading blood biomarker for AD, pTau‐217, demonstrates high sensitivity and specificity for identifying individuals at risk for AD dementia, or who likely have underlying AD pathology. However, blood biomarker levels are influenced by some co‐morbidities, and sources of unexpected fluctuations are not well understood (Brum et al, 2023). Given their potential of causing misclassifications, characterizing these fluctuations is crucial given their increasing use in a biomarker‐supported AD diagnosis and as eligibility tests for clinical trial enrollment.

**Method:**

Draw‐10 is an observational research study exploring fluctuations in blood biomarkers over a short period and potential associated drivers of these fluctuations. Draw‐10 will enroll approximately 100 participants aged 50‐80 years old. Participant population will include individuals with normal cognition and MCI based on a screening MoCA of ≥18. Over 9 weeks, participants will provide 10 plasma samples and complete questionnaires at each visit regarding their weekly alcohol use and exercise behaviors. pTau‐217 assays of different designs (*n*‐terminal, brain‐derived, and ratios) will be assessed. Following the conclusion of sample collection, participants will have the option to learn their pTau‐217 results from a single validated result via a telehealth visit.

**Result:**

Enrollment began in January 2025 and is expected to close in June 2025. Recruitment, screening, and enrollment data will be presented along with participant demographics. Blood biomarker analysis will be performed in one batch. Analysis will be randomized within each participant and performed blinded to any participant information. Results will estimate the analytical and biological variation for *p*‐tau217 assays, reference change values, the frequency of major fluctuations in blood biomarkers, and if this can be attenuated by different assay immunoassay designs or ratios.

**Conclusion:**

The Draw‐10 study addresses the need for further understanding the biological variation of AD blood biomarkers and for identifying potential sources of fluctuations in their levels. These results may improve biomarker‐supported clinical decisions, prevent inappropriate blood biomarker based screening decisions of research participants and determining the minimum number of samples required for a reliable *p*‐Tau217 measurement.

